# Bone Morphogenetic Protein 7 Improves Wound Healing in Diabetes by Decreasing Inflammation and Promoting M2 Macrophage Polarization

**DOI:** 10.3390/ijms26052036

**Published:** 2025-02-26

**Authors:** Jessica Da Silva, Ana Figueiredo, Yu-Hua Tseng, Eugenia Carvalho, Ermelindo C. Leal

**Affiliations:** 1 Doctoral Program in Experimental Biology and Biomedicine (PDBEB), Institute of Interdisciplinary Research, University of Coimbra, 3004-504 Coimbra, Portugal; jessicasilva@cnc.uc.pt; 2 CNC-UC—Center for Neuroscience and Cell Biology, University of Coimbra, 3004-504 Coimbra, Portugal; ana.figueiredo@gimm.pt (A.F.); ecarvalh@cnc.uc.pt (E.C.); 3 CIBB—Centre for Innovative Biomedicine and Biotechnology, University of Coimbra, 3030-788 Coimbra, Portugal; 4 Joslin Diabetes Center, Harvard Medical School, Boston, MA 02215, USA; 5 Institute of Interdisciplinary Research, University of Coimbra, 3004-504 Coimbra, Portugal

**Keywords:** angiogenesis, bone morphogenetic protein 7, collagen deposition, diabetic wound healing, inflammation, oxidative stress, tissue remodeling

## Abstract

Diabetic foot ulcers (DFUs) are a devastating complication of diabetes, presenting limited treatment success rates due to their complex pathophysiology. Bone morphogenetic protein 7 (BMP7) confers tissue protective and regenerative functions, but its potential role in diabetic wound healing is unknown. The aim of this study was to investigate the effects of topical BMP7 treatment in wound healing using a streptozotocin-induced diabetic mouse model. The expression of markers of wound healing progression were detected using RT-PCR or immunohistochemistry. Overall, BMP7 improved wound closure, as well as maturation of granulation tissue and collagen deposition, as evidenced by hematoxylin and eosin and Masson’s trichrome histological analysis. The expression of inflammatory markers (IL-6, TNF-α) and matrix metalloproteinase-9 were decreased in BMP7-treated wounds, together with the number of pro-inflammatory M1 macrophages and T lymphocytes. The number of anti-inflammatory M2 macrophages was increased in BMP7-treated wounds. Moreover, BMP7 decreased oxidative stress and increased Ki67^+^ cells and CD31^+^ cells, indicating induced proliferation and angiogenesis in the wound bed compared to the control wounds. Finally, BMP7 activated the ERK pathway and suppressed the p38 pathway in diabetic wounds. Together, our data suggest that BMP7 enhanced skin wound healing in diabetes by decreasing local inflammation and oxidative stress, which promoted a regenerative environment for collagen deposition, wound maturation, cell proliferation, and angiogenesis. These findings underline BMP7 as a potential therapeutic agent for the treatment of skin wounds in diabetes.

## 1. Introduction

Diabetes mellitus is one of the most prevalent chronic metabolic diseases worldwide. Based on the International Diabetes Federation (IDF) Diabetes Atlas 2021, over 500 million people have diabetes globally, and this number is estimated to increase to over 700 million by 2045 [1]. Diabetic foot ulceration (DFU) is one of the most common and debilitating complications of diabetes and it is associated with delayed wound healing. Approximately 19 to 34% of patients with diabetes will develop DFU, which often requires prolonged hospitalizations for its management [2,3]. Additionally, more than half of DFUs become infected, requiring subsequent amputation in 20% of cases facing moderate to severe infections [2,3]. The pathogenesis of chronic non-healing DFUs is not yet fully understood and therefore the development of more effective treatment for chronic diabetic wounds is imperative.

Wound healing involves a coordinated sequence of cellular and molecular events, consisting in four stages: hemostasis, inflammation, proliferation, and remodeling [4,5,6,7]. However, under diabetes conditions, these healing phases become stalled, particularly in the early inflammatory phase, predisposing patients to chronic diabetic foot ulcers [8]. In contrast to normal wounds, diabetic wounds are characterized by persistent inflammation [8,9], increase infiltration of inflammatory cells, and expression of pro-inflammatory cytokines, which is associated with the overproduction of reactive oxygen species (ROS), causing significant tissue damage [8,10,11,12]. Macrophages are key players in wound repair, and, in our previous studies, we found that the modulation of the macrophage phenotype by neuropeptides improve diabetic wound healing [10]. Pro-inflammatory M1 macrophages are important in the initial phases of wound healing and their differentiation, in later phases, into anti-inflammatory M2 macrophages allow for the progression of wound healing [13,14]. In addition, diabetic conditions lead to impaired angiogenesis, cell migration and proliferation, as well as the degradation of the extracellular matrix (ECM) [12,15].

Bone morphogenetic proteins (BMPs) belong to the transforming growth factor-beta (TGF-β) superfamily and were found to be important in the regeneration of bones, but nowadays are involved in several other functions [16]. BMP7 is one the key players in bone formation by inducing mesenchymal cells differentiation into osteoblasts, but it is also associated with other important roles, such as cell proliferation and differentiation, and extracellular matrix proteoglycan and type II collagen synthesis [17]. In addition, BMPs have important functions in immune-mediated disorders due to their impact on the immune system in systemic chronic diseases such as liver disease, rheumatoid arthritis, and atherosclerosis [18,19,20,21]. BMP7 activates two major signaling pathways: (1) Canonical/Smad dependent and (2) Non-canonical/Smad independent pathway [22]. In the canonical pathway, BMP7 activates Smad-1/5/8, which complexes with Smad-4 and translocate the signal. In the non-canonical pathway, mitogen-activated protein kinase (MAPK)-dependent pathways are activated [23]. BMP7 is expressed by several tissues, including the skin [22,23]. Lower levels of BMP7 are also associated with various diseases, including osteoporosis, cardiovascular diseases and diabetes [21,24,25,26]. Several studies have shown that the anti-inflammatory effect attributed to BMP7 is achieved by reducing the expression of pro-inflammatory cytokines, such as tumor necrosis factor-alpha (TNF-α); interleukin (IL)-6, IL-8, and IL-1β; and chemokine monocyte chemoattractant protein-1 (MCP-1), as well as by enhancing anti-inflammatory M2 macrophage differentiation [27,28].

Given the lack of studies assessing the potential protective actions of BMP7 in diabetic complications, particularly in DFU, we sought to investigate whether this treatment would confer an improvement on wound healing by using a streptozotocin (STZ)-induced diabetes animal model of wound healing. To our knowledge, this is the first study to determine the therapeutic potential of BMP7 in diabetic wound healing.

## 2. Results

### 2.1. BMP7 Treatment Accelerates Wound Healing in Diabetic Mice

**Figure 1:**
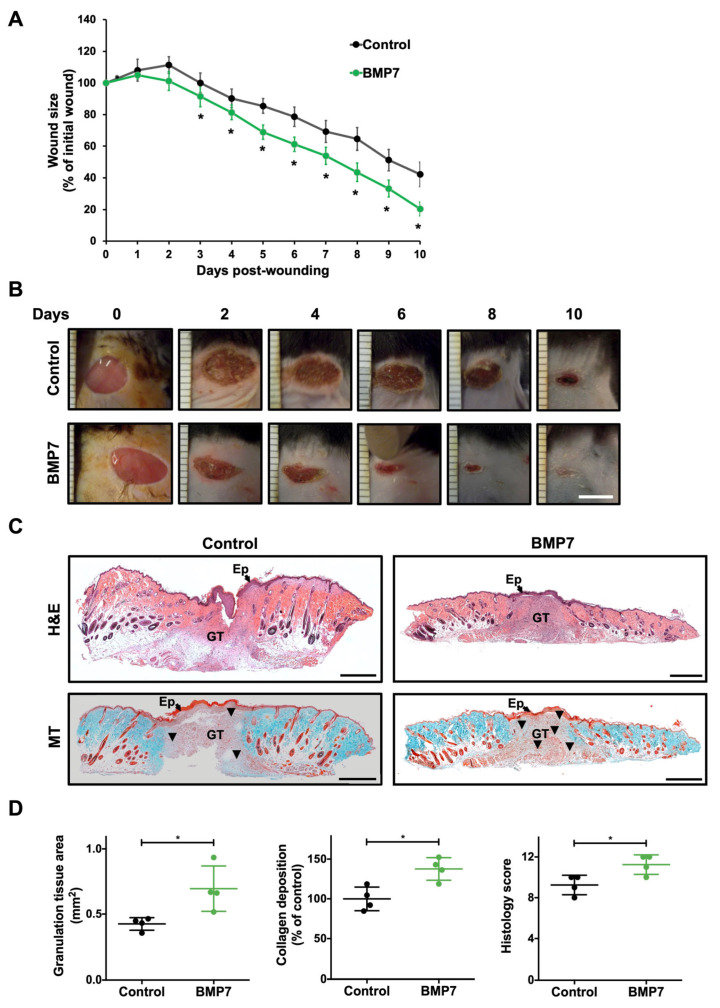
BMP7 treatment promoted wound healing progression in mice with diabetes. (**A**) Quantification of wound closure (% of initial wound area) in diabetic animals up to 10 days post-injury. (**B**) Representative images of wound size for each experimental group at days 0, 2, 4, 6, 8, and 10. Scale bar—5 mm. (**C**) Representative images of hematoxylin and eosin (H&E), and Masson’s trichrome (MT) staining of skin wounds at day 10 post-wounding. Scale bars—500 µm. Black triangles indicate increased collagen deposition in wounded area. Ep: epidermis; GT: granulation tissue. (**D**) Quantification of the granulation tissue area, collagen deposition, and histology score. Data are presented as mean ± SD (n = 4 animals for each experimental group). Statistical analysis was conducted using Student’s *t*-test. * *p* < 0.05. The skin wounds were treated with saline (control) or human recombinant protein BMP7.

To investigate the effects of BMP7 on skin wound healing in diabetes, we used an excisional wound model in mice with STZ-induced diabetes. We assessed the wound healing progression by measuring the percentage of wound area relative to the initial wound area (Figure 1A,B). The results showed a significant decrease in the wound size in the BMP7-treated group at days 3 to 10 post-wounding, compared to the control (saline) group (*p* < 0.05, n = 4 animals, 8 wounds).

### 2.2. BMP7 Treatment Promotes More Mature Wound Healing

In hematoxylin and eosin (H&E)-stained sections, at day 10 post-wounding, the BMP7-treated wounds exhibited a significant increase in the formation of granulation tissue with a dense structure at the wound site, compared to the saline-treated wounds (Figure 1C,D). The granulation tissue area in BMP7-treated wounds was significantly higher than the one from saline-treated wounds (0.7 ± 0.1 mm^2^ and 0.4 ± 0.04 mm^2^, respectively, *p* < 0.05). Masson’s trichrome (MT) staining was performed to detect the collagen deposition at day 10 post-wounding within the granulation tissue (Figure 1C,D). The collagen deposition was significantly elevated in the BMP7-treated group to 137.6 ± 14.2% of control (*p* < 0.05). To evaluate the maturation of the wound, we used a histology scoring system analyzing the H&E and MT sections, as described previously [29] (Figure 1D). BMP7-treated wounds had a significantly higher histological score than the saline-treated wounds (11.3 ± 0.9 and 9.3 ± 0.9, respectively, *p* < 0.05).

### 2.3. BMP7 Treatment Decreases Inflammatory Markers and MMP-9 in Wounds of Diabetic Mice

**Figure 2:**
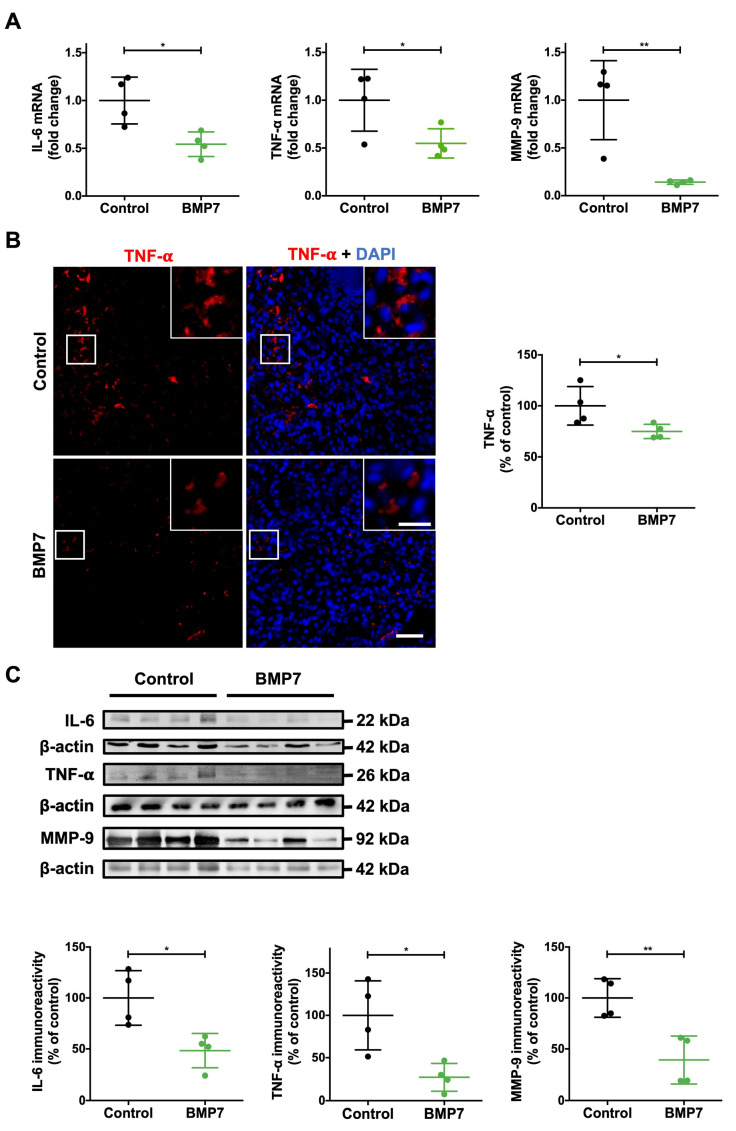
BMP7 decreased inflammatory markers in skin wounds at day 10 post-injury in mice with diabetes. (**A**) Quantification of IL-6, TNF-α, and MMP-9 mRNA levels by quantitative PCR. (**B**) Representative images of TNF-α immunohistochemistry. Scale bars—20 µm (picture inset) and 50 µm. (**C**) Representative images and quantification of IL-6, TNF-α, and MMP-9 protein expression levels. Data are presented as mean ± SD (n = 4 animals for each experimental group). Statistical analysis was conducted using Student’s *t*-test. * *p* < 0.05, ** *p* < 0.01. The skin wounds were treated with saline (control) or human recombinant protein BMP7.

To evaluate the level of inflammation in the wounds, the gene and protein expressions of the inflammatory markers IL-6 and TNF-α were determined (Figure 2). Additionally, matrix metalloproteinase-9 (MMP-9), known to be highly expressed in the inflammation phase of wounds [10], was therefore also evaluated. The mRNA levels of IL-6 and TNF-α were significantly decreased in wounds of diabetic animals treated with BMP7 compared with wounds treated with saline (IL-6: 0.54 ± 0.13 fold change of saline treatment, *p* < 0.05; TNF-α: 0.55 ± 0.15 fold change of saline treatment, *p* < 0.05) (Figure 2A). Moreover, BMP7 treatment was able to significantly decrease mRNA levels of MMP-9 compared with wounds treated with saline (0.14 ± 0.02 fold change of saline treatment, *p* < 0.01) (Figure 2A).

Similarly, the immunohistochemistry analysis of TNF-α protein levels showed a significant decrease in BMP7-treated wounds, compared to control wounds (TNF-α: 74.9 ± 7.0% of control, *p* < 0.05) (Figure 2B).

Moreover, the protein expression analysis further confirmed these results, demonstrating a significant decrease in the inflammatory markers IL-6 and TNF-α protein levels in BMP7-treated wounds, compared to control wounds (IL-6: 48.3 ± 16.8% of control, *p* < 0.05; TNF-α: 27.1 ± 16.1% of control, *p* < 0.05) (Figure 2C). Likewise, BMP7 treatment further decreased the MMP-9 protein expression levels to 39.2 ± 23.4% of control (*p* < 0.01) (Figure 2C). These data suggest that BMP7 decreases the persistent inflammatory state in diabetic wounds and the damaging effect of MMP-9 in the wound extracellular matrix.

### 2.4. BMP7 Treatment Decreases Inflammatory Cells at the Wound Site

We next investigated whether BMP7 treatment regulated inflammation by modulating inflammatory cell dynamics. The imbalance of immune cells in wound healing will lead to the deterioration of the immune microenvironment, and the wound will be stagnant in the inflammatory stage, which will further hinder the transition of wound healing from inflammation to proliferation and remodeling and impair wound repair.

**Figure 3:**
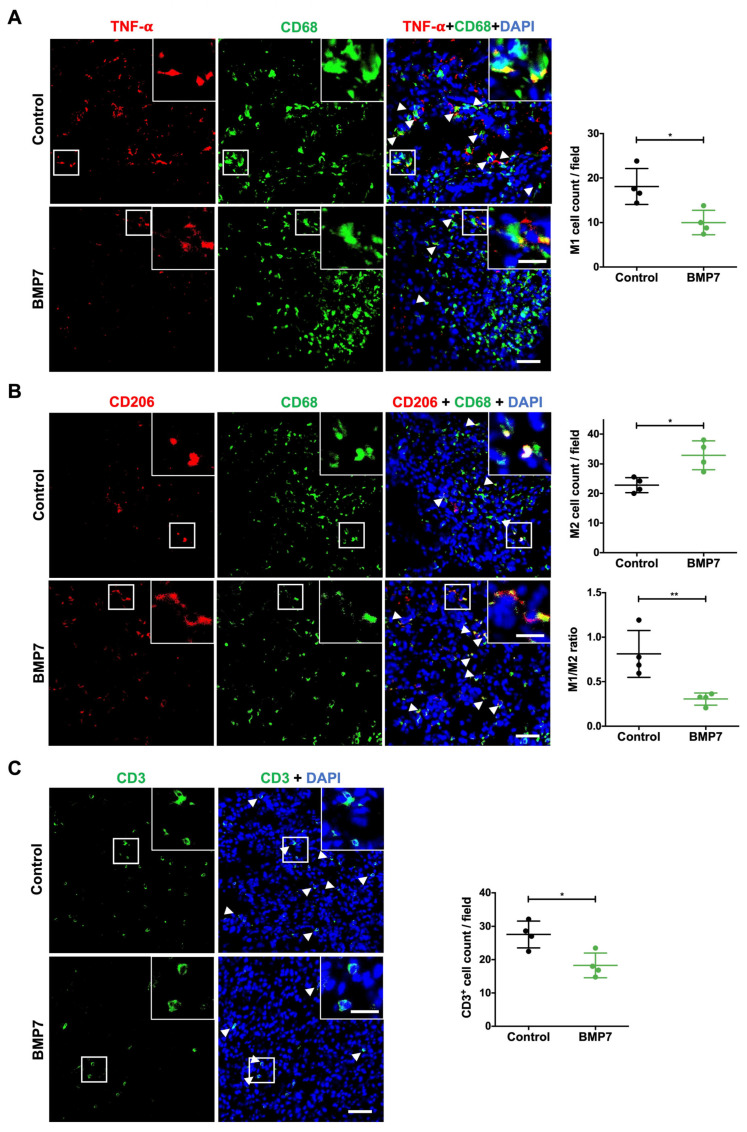
Effect of BMP7 treatment in the number of immune cells in the wound site at day 10 post-injury. (**A**) Representative images of M1-like macrophages and respective quantification. M1-like macrophage phenotype was identified by the expression of CD68 (green) and TNF-α (red). The nuclei were stained with DAPI (blue). Arrows indicate M1-like macrophages. (**B**) Representative images of M2-like macrophages and respective quantification, as well as determination of M1/M2 ratio. M2-like macrophage phenotype was identified by the expression of CD68 (green) and CD206 (red). The nuclei were stained with DAPI (blue). Arrows indicate M2-like macrophages. (**C**) Representative images of CD3^+^ cells and respective quantification. T cells are identified by the expression of CD3 (green). The nuclei were stained with DAPI (blue). Arrows indicate CD3^+^ cells. Scale bars—20 µm (picture inset) and 50 µm. Data are presented as mean ± SD (n = 4 animals for each experimental group). Statistical analysis was conducted using Student’s *t*-test. * *p* < 0.05, ** *p* < 0.01. The skin wounds were treated with saline (control) or human recombinant protein BMP7.

Macrophages, the major inflammatory cells present in wounds, are the core cells involved in the wound healing process. In normal wound healing, pro-inflammatory M1 macrophages dominate the wound healing site in the inflammatory phase and then begin to transition to M2 macrophages, which promote tissue regeneration. Thus, we evaluated the number of M1 and M2 macrophages. In this study, we used CD68 to mark macrophages and particularly TNF-α to mark M1-like macrophages and CD206 to mark M2-like macrophages. The number of M1-like macrophages in the control group (saline) was significantly higher than in the BMP7-treated group (18.1 ± 4.0 and 10.0 ± 2.7, respectively, *p* < 0.05) at day 10 post-wounding (Figure 3A). Additionally, as shown in Figure 3B, there was an increase in the number of M2-like macrophages with the BMP7 treatment on day 10 post-wounding, compared to saline treatment (32.9 ± 4.8 and 22.8 ± 2.6, respectively, *p* < 0.05). In addition, the M1/M2 ratio, that infer to the inflammatory state of the tissue, was higher in the saline treatment compared to the BMP7 treatment (0.8 ± 0.2 and 0.3 ± 0.06, respectively, *p* < 0.01) (Figure 3B).

T cells, also called T lymphocytes, are also very important in the inflammatory phase and contribute to the inflammatory imbalance in diabetic wounds. Thus, we evaluated the number of T cells in wounds with the marker CD3. We found that BMP7 treatment decreased the number of T cells compared to saline-treated wounds at day 10 post-wounding (18.3 ± 3.7 and 27.6 ± 4.0, respectively, *p* < 0.05) (Figure 3C). Together, these data suggested that BMP7 decreases the inflammatory environment by reducing the number of inflammatory cells.

### 2.5. BMP7 Treatment Decreases Oxidative Stress and Promotes Cell Proliferation and Angiogenesis

**Figure 4:**
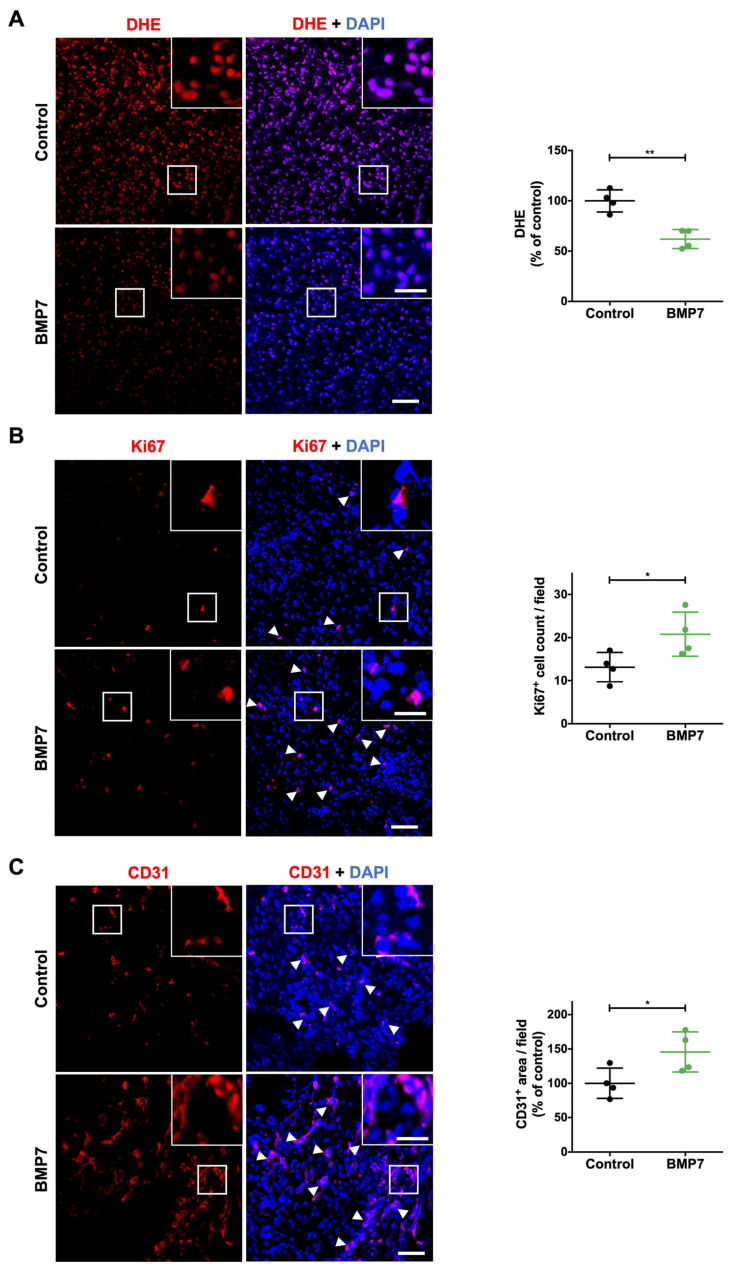
Effect of BMP7 treatment on oxidative stress, proliferation, and angiogenesis in the wound site at day 10 post-injury. (**A**) Representative images and quantification of dihydroethidium (DHE) assay (red) for oxidative stress, (**B**) Ki67^+^ (red) cells for proliferation, and (**C**) CD31^+^ (red) endothelial cells for angiogenesis. The nuclei were stained with DAPI (blue). Arrows indicate Ki67^+^ or CD31^+^ cells. Scale bars—20 µm (picture inset) and 50 µm. Data are presented as mean ± SD (n = 4 animals for each experimental group). Statistical analysis was conducted using Student’s *t*-test. * *p* < 0.05, ** *p* < 0.01. The skin wounds were treated with saline (control) or human recombinant protein BMP7.

Along with increased inflammation, the excessive production of ROS in diabetic wounds can compromise cell proliferation and angiogenesis. Thus, we used the dihydroethidium (DHE) staining to evaluate ROS levels. Moreover, we used the marker Kiel 67 (Ki67) for cell proliferation and the CD31 marker for angiogenesis. The levels of ROS were significantly decreased in BMP7-treated wounds compared to saline-treated wounds at day 10 post-wounding (62.0 ± 9.4% of control, *p* < 0.01) (Figure 4A). The number of Ki67^+^ cells was significantly higher in BMP7-treated wounds compared to those treated with the control (20.8 ± 5.1 and 13.1 ± 3.4, respectively, *p* < 0.05) (Figure 4B). Similarly, angiogenesis, represented by the area of CD31^+^ cells, was increased in BMP7-treated wounds compared to saline-treated wounds (145.6 ± 29.4% of control, *p* < 0.05) (Figure 4C). Taken together, these data suggested that BMP7 decreases excessive oxidative stress in diabetic wounds, likely promoting cell proliferation and angiogenesis.

### 2.6. BMP7 Decreases the p38 Pathway and Activates the ERK and AKT Pathways

**Figure 5:**
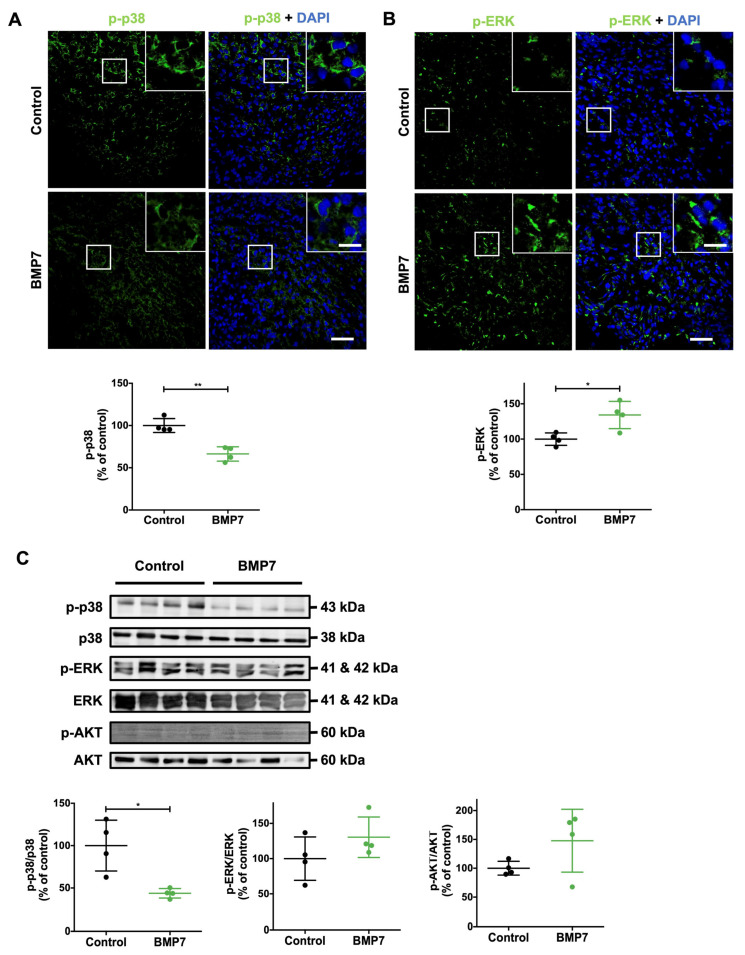
BMP7 treatment decreased the p-p38 pathway and increased p-ERK in the wound site at day 10 post-injury. (**A**) Representative image and quantification of p-p38 (green), and (**B**) p-ERK (green) immunohistochemistry. The nuclei were stained with DAPI (blue). Scale bars—20 µm (picture inset) and 50 µm. (**C**) Immunoreactivity quantification of p-p38/p38, p-ERK/ERK, and p-AKT/AKT protein expression levels. Data are presented as mean ± SD (n = 4 animals for each experimental group). Statistical analysis was conducted using the student’s *t*-test. * *p* < 0.05, ** *p* < 0.01. The skin wounds were treated with saline (control) or human recombinant protein BMP7.

Several signaling pathways involved in wound healing can be regulated by BMP7 treatment. Here, we evaluated the activation of the p38, ERK, and AKT pathways through the expression levels of the corresponding phosphorylated proteins by immunohistochemistry. The BMP7 treatment significantly decreased the expression of phosphorylated p38 (p-p38) compared to the saline treatment (66.3 ± 8.5% of control, *p* < 0.01) (Figure 5A). In contrast, the BMP7 treatment significantly increased phosphorylated ERK (p-ERK) expression levels compared to the saline treatment (134.2 ± 19.3% of control, *p* < 0.05) (Figure 5B).

Similarly, p-p38/p38 protein expression levels in BMP7-treated wounds were significantly decreased compared to saline-treated wounds (43.7 ± 5.6% of control, *p* < 0.05) (Figure 5C). Additionally, both p-ERK/ERK and p-AKT/AKT protein expression levels tended to increase upon BMP7 treatment, compared to the saline treatment, yet without reaching significance (p-ERK: 130.3 ± 28.8% of control; p-AKT: 147.7 ± 54.5% of control) (Figure 5C).

## 3. Discussion

DFU is one of the most common complications of diabetes and, due to its complex pathophysiology, effective treatment options are limited. Therefore, investigating novel therapeutic methods is important for improving treatment strategies for patients with DFUs. BMP7 has been reported to promote bone repair and wound healing in the cornea and thermal injury models [22,30]. In the present study, recombinant human BMP7 was topically added to skin wounds of diabetic mice, and the effect on wound healing was investigated. To the best of our knowledge, the present study is the first to report that BMP7 treatment promotes wound healing in a diabetic mouse model. This provides a novel insight into the role of BMP7 in skin wound healing in diabetes and it indicates that BMP7 may represent a potential therapeutic target for the treatment of DFUs.

The formation and resolution of the granulation tissue is impaired in diabetes, being responsible for the delayed healing progression. Here, we found that BMP7 was able to promote wound closure with the formation of a mature granulation tissue with increased collagen deposition. This BMP7 effect is very important since the wounds of patients with diabetes showed to have a loose extracellular matrix in the wound bed, which impairs cellular functions such as migration and proliferation, important for wound healing progression [31]. Despite the increasing understanding of BMP7 signaling transduction and regulation, there are also controversial results in its efficiency. BMP7 has been reported to have an anti-fibrosis effect in several organs with potential application in the treatment of fibrotic diseases [32]. Moreover, BMP7 was shown to inhibit the scar formation via the inhibition of the TGFβ pathway by reducing excessive collagen deposition in a mouse model of skin thermal injury [22]. Nevertheless, other reports showed that BMP7 can induce the TGFβ pathway and induce collagen deposition [30,33]. In this work, we found that BMP7 can induce collagen deposition and promote a mature granulation tissue in the wound bed. This can be related to the several signaling pathways activated by BMP7. The canonical pathway, via Smad signaling pathway, has an important role in collagen metabolism and has been confirmed as one of the most extensively studied signaling pathways in scar formation [20,22]. Here, since we showed an increase in collagen deposition with BMP7 treatment, we studied the non-canonical pathway and showed that the ERK pathway was increased in the wound bed, which is in accordance with promoting collagen deposition, cell migration, and wound healing [30,34]. The opposing effects of BMP7 can also depend in the regulation of BMP7 levels and in the amount used for the treatment. In fact, Guo et al. showed that the treatment with 100 μg BMP7 prevented the collagen deposition induced by the skin thermal injury model [22]. In this work, we used 0.5 μg BMP7 for the wound treatment which promoted collagen deposition in a diabetic wound healing animal model, known to show a decrease in collagen deposition [35,36]. Further studies are needed to understand how BMP7 can promote collagen deposition, possibly via the TGFβ pathway, since BMP7 in certain concentrations can activate the TGFβ pathway [30,33].

After skin injury, an inflammatory response is important to the onset of wound healing followed by the resolution of inflammation and tissue regeneration. In chronic wounds, the failure in the resolution of inflammation contributes to the delayed healing progress. A persistent inflammatory response is found in both patients and animal models of diabetes [37,38,39,40]. The presence of inflammatory cells leads to a persistent release of pro-inflammatory cytokines such as TNF-α and IL-6, among others [10,41]. In DFU, pro-inflammatory M1 macrophages dominate the wound setting and perpetuate inflammation, whereas in normal wounds, M1 macrophages are progressively replaced by anti-inflammatory M2 macrophages to promote tissue regeneration [41,42]. Furthermore, neutrophils and lymphocytes are also responsible for wound healing impairment in diabetes [37,40,43]. In fact, treatments that modulate inflammation are potential therapeutic options for the resolution of chronic wounds, particularly in diabetes. Several studies suggest that BMP7 has anti-inflammatory activity by suppressing pro-inflammatory cytokine production and promoting M2 macrophage polarization both in vitro and in vivo [16,27,28]. However, the role of BMP7 in inflammatory mediation of diabetic wound healing is not known. T lymphocytes and M1 macrophages play a key role in the triggering and maintenance of inflammation in diabetic wound healing [10,44]. The results of our study demonstrated that BMP7 reduced the diabetes-induced elevated number of T lymphocytes and pro-inflammatory M1 macrophages in wounds of diabetic mice. These effects correlated with the suppression of the expression of pro-inflammatory cytokines, IL-6, and TNF-α, known to promote chronic DFU [39,45], in BMP7-treated wounds, suggesting an anti-inflammatory role of BMP7. Furthermore, we found that MMP-9 expression was increased in wounds of diabetic mice and this effect was suppressed by the BMP7 treatment. It is known that a prolonged inflammatory response promotes the secretion of proteases such as MMP-9 by inflammatory cells, which generally destroy the wound microenvironment [10,46], leading to a weak granulation tissue and collagen degradation. Elevated levels of MMP-9 have been associated with poor wound outcomes and have been considered as therapeutic targets for DFU [47,48,49].

Moreover, the number of M2 macrophages and the M1/M2 ratio were evaluated to elucidate the immunomodulatory effect of BMP7 in wounds of animals with diabetes. BMP7 treatment increased the number of M2 macrophages in diabetic wounds and decreased the M1/M2 ratio, which is indicative of macrophage polarization and decreased levels of wound inflammation [10,50]. These results suggest that the anti-inflammatory effect of BMP7 is related with the ability to enhance the polarization to M2 macrophages, leading to a decrease in the expression of pro-inflammatory cytokines. This effect was also observed in other studies, where BMP7 promoted the differentiation of a human monocyte cell line, THP1, into M2 macrophages, as well as in inflammatory disease animal models such as atherosclerosis and inflammatory bowel disease [16,27,28]. Furthermore, these studies in animal models of inflammatory disease suggest that BMP7 reduces the expression of pro-inflammatory cytokines and promote M2 macrophage polarization by suppressing the activation of the p38 pathway, while increasing the activation of ERK pathways [16,19,28]. In fact, our study showed that BMP7 decreased p38 phosphorylation and increased ERK phosphorylation in wounds of diabetic mice. In addition, BMP7 is reported to support a protective anti-inflammatory effect through PI3K/Akt pathway activation [51,52]; however, in diabetic wounds, BMP7 was not able to significantly increase the phosphorylation of AKT. It will be relevant to further assess the role of BMP7 on inflammatory responses, particularly evaluating the effect of BMP7 in the expression of anti-inflammatory markers.

Diabetic wounds are further characterized by the presence of elevated levels of ROS essentially due to the persistent inflammatory condition [53,54]. Although the production of ROS is important in wound healing [55,56], excessive release of ROS can lead to cell and tissue damage and delayed wound healing in DFU [57]. Importantly, pathological oxidative stress can alter ECM structure and function [58]. We showed that the BMP7 treatment decreased oxidative stress in diabetic wounds, which could contribute to the maturation of granulation tissue. Likewise, other studies demonstrated that BMP7 has an antioxidative role. BMP7 protected cultured neurons from oxidative stress induced by beta amyloid [59]. Additionally, BMP7 decreased oxidative stress in a diabetic chronic kidney disease animal model [60]. In accordance with our study, this antioxidative activity of BMP7 might be exerted through the suppression of the p38 pathway [60]. Our results also demonstrated that BMP7 promoted cell proliferation and angiogenesis, which are known to be dysfunctional in diabetic wounds due to the elevated oxidative stress and inflammatory condition [61]. In fact, BMP7 induces proliferation of cardiomyocytes and corneal epithelial cells, promoting cardiovascular regeneration and corneal wound healing, respectively, through the activation of the ERK signaling pathway [30]. Additionally, BMP7 promotes the angiogenic potential of HUVECs and induces vascularization in pulp-like tissue regeneration [62].

One limitation of this study was the use of animal models of type 1 diabetes. However, there are no differences in the pathophysiology of diabetic foot ulceration between human type 1 and type 2 diabetes. Altogether, topical treatment with BMP7 has the potential to promote healing in diabetic ulcers. However, BMP7 effects can be dose-dependent, particularly in promoting or reducing collagen deposition [22,30,33], as well as in inducing osteogenic differentiation in skin fibroblasts [63]. Therefore, caution will be needed with the dosage to be used, and more pre-clinical studies will be important to fully understand the potential of BMP7 for the treatment of these chronic non-healing complex wounds.

## 4. Materials and Methods

### 4.1. Animals

Eight-week-old male C57BL/6J mice were purchased from Charles River Laboratories (Paris, France) and housed in certified local facilities at normal room temperature under a 12 h light/dark cycle, with free access to water and food. All the experimental protocols involving animals were approved by the animal research ethics committee of the Center for Neuroscience and Cell Biology and the Faculty of Medicine of the University of Coimbra (ORBEA_213_2019/28082019) and by the national (Directorate-General for Food and Veterinary of the Portuguese Ministry of Agriculture) research ethical committee. Also, the animal protocols were in accordance with the European Directive 2010/63/EU and the Portuguese Decree-law (113/2013) for the use of animals for scientific purposes.

### 4.2. Diabetic Animal Model of Wound Healing

Diabetes was induced in mice by intraperitoneal (IP) administration of 50 mg/kg of streptozotocin (STZ) for five consecutive days, as previously described [10]. One week after the induction of diabetes, blood glucose levels were measured to confirm the diabetic state. Mice with blood glucose levels above 250 mg/dL were considered diabetic. Mice were treated with 0.1–0.2 units of NPH insulin (subcutaneous—s.c.) as needed, to prevent weight loss. The animals were kept diabetic for 6 weeks prior to the wound healing experiments and the weight and blood glucose levels were measured before randomly assigning the animals to the different treatment groups (Table 1). Analgesia was given before wounding (0.05 mg/kg buprenorphine, s.c.) and every 8 h up to 24 h after wounding (0.1 mg/kg buprenorphine, s.c.). The animals were then anesthetized with 5% isoflurane, and maintained with 2.5%, combined with oxygen (0.5 L/min). Afterwards, the dorsal hair of the mice was removed, skin was sterilized with a povidone–iodine antiseptic solution (Betadine), and two full-thickness wounds were made in each mouse with a 6 mm biopsy punch tool. The mice were divided into two experimental groups according to the different wound treatments: BMP7—0.5 μg human recombinant BMP7 (R&D Systems, Bristol, UK) diluted in a sterile saline 0.9% NaCl solution containing 4 mM HCl (n = 4), or control—saline 0.9% NaCl solution containing 4 mM HCl (n = 4). The dose of BMP7 was selected based in previous in vivo studies in mice that received 0.72 μg systemically of BMP7 per day [64]. Since, this dose was not toxic for the animals and it is known that BMP7 can enhance the osteogenic differentiation of fibroblast cells in skin [63] in higher doses, we therefore chose to apply 0.5 μg/wound/day topically and no toxic effect or osteogenic differentiation was observed. The treatments were applied topically in each wound daily during the 10-day course of the wound healing period. The wound size was daily monitored and measured by acetate tracing and quantified with Fiji software (version 2.14.0/1.54f, NIH Image, Bethesda, MD, USA). At day 10 after wounding, mice were anesthetized with ketamine/xylazine (100/10 mg/kg, intraperitoneally) and euthanized by cervical dislocation. The wounded skin was harvested and cryopreserved in optimal cutting temperature (OCT) gel (VWR, Carnaxide, Portugal) at −80 °C, or fixed in 4% paraformaldehyde (PFA) (Sigma, Steinheim, Germany) in phosphate-buffered saline (PBS) at 4 °C, for further analysis.

### 4.3. Histological Analysis

After fixation (4% PFA) at 4 °C for 48 h, the wounded skin was included in paraffin. Paraffin-embedded skin sections (5 µm thickness) were stained with hematoxylin and eosin (H&E) (Sigma, Germany) and Masson–Goldner’s trichrome (MT) (Carl Roth, Karlsruhe, Germany) kits, according to the manufacturers’ protocols, to evaluate the wound structure and collagen deposition, respectively. The wound image sections, obtained with combinations of images (100× magnification), were acquired using a Carl Zeiss Axio Imager Z2 upright widefield microscope (Carl Zeiss, Oberkochen, Germany).

A histology scoring system was used to evaluate the effect of the BMP7 treatment in wound healing progression by using H&E- and MT-stained skin sections. This system was based on a previously validated histological scoring method for murine skin wounds [29].

### 4.4. Quantitative Real-Time PCR

Total RNA was isolated from skin tissue using a RNeasy Mini Kit (Qiagen, Hilden, Germany), following the manufacturer’s instructions, and concentration was determined by OD260 measurement using the NanoDrop spectrophotometer (Thermo Scientific, Waltham, MA, USA). cDNA was prepared from 1 μg of RNA using the High-Capacity cDNA Reverse Transcription kit (Applied Biosystems, ThermoFisher Scientific, Porto Salvo, Portugal). Briefly, 2 μL of 10× RT Buffer, 0.8 μL of 25× dNTP Mix, 2 μL of 10× RT random primers, 1 μL of MultiScribe Reverse Transcriptase, and 4.2 μL of nuclease free H_2_O were added to 10 μL of RNA (1 μg) sample. Quantitative real-time PCR was performed using with a Bio-Rad MyCycler iQ5 real-time PCR thermal cycler (Bio-Rad, Lisboa, Portugal). For each reaction, 10 μL volume were used containing 2.5 μL cDNA, 5 μL 2× PerfeCTa^®^ SYBR^®^ Green FastMix (VWR, Portugal), 1 μL of each primer (250 nM), and 0.5 μL of H_2_O PCR grade. The primer sequences (Table 2) were obtained from Integrated DNA Technologies (Coralville, IA, USA). The gene expression was determined by the ΔΔCt method of relative quantification obtained as 2^(−ΔΔCt) and normalized to the TATA box binding protein (Tbp) gene. Data are presented as the fold change over the gene expression of the control sample.

### 4.5. Immunohistochemistry

Skin cryosections (10 µm thickness) were fixed in ice-cold acetone for 10 min and permeabilized at RT with PBS with 1% Tween (PBS-T) and 0.2% Triton X-100 for 30 min. Subsequently, the samples were blocked with 50 µL of 10% goat serum for 30 min at RT. Then, the samples were placed in a humidified chamber and incubated overnight at 4 °C with the primary antibodies: rabbit anti-CD68 (1:100, Abcam, Cambridge, UK) and rat anti-TNF-α (1:200, AbD Serotec, Oeiras, Portugal); rat anti-CD206 (1:200, Santa Cruz, Santa Cruz, USA); rat anti-CD31(1:200, PECAM-1, Merck Millipore, Darmstadt, Germany); rabbit anti-CD3 (1:100, Abcam, UK); rabbit anti-IL6 (1:100, Abcam, UK); rabbit anti-pp38 (1:100, Abcam, UK); rabbit anti-pERK (1:100, Cell Signaling, Danvers, MA, USA); and rabbit anti-pAKT (1:100, Cell Signaling, USA). The samples were then incubated at RT for 1 h, with DAPI (1:1000) for nuclei staining and the secondary antibody, anti-rat (1:500, conjugated to Alexa Fluor 568, Invitrogen, Waltham, MA, USA), and anti-rabbit (1:500, Alexa Fluor 468 conjugated, Invitrogen, USA). The 3–5 random images at the wound site were obtained using the Carl Zeiss LSM 710 confocal microscope with a 200× magnification and acquired using Zen Blue software (version 3.8). The number of cells, area, or fluorescent staining intensity was analyzed using Fiji software (NIH Image, USA).

### 4.6. Western Blot Analysis

The skin samples were homogenized in radioimmunoprecipitation assay (RIPA) buffer (50 mMTris–HCl buffer pH 7.5, 150 mM NaCl, 1% Triton X-100, 0.5% sodium deoxycholate, 0.1% sodium dodecyl sulfate, 5 mM ethylene glycol tetraacetic acid, protease inhibitor cocktail, phosphatase inhibitor cocktail, and 1 mM dithiothreitol). The protein concentration was measured using the bicinchoninic acid (BCA) method (Pierce^®^ BCA Protein Assay Kit, Thermo Scientific). The skin tissue lysates were denatured at 95 °C for 5 min in 6× sample buffer (0.35 M Tris–HCl, pH 6.8, 30% glycerol, 10% sodium dodecyl sulfate, 0.6 M dithiothreitol, 0.03% bromophenol blue). Equal amounts of protein (30 or 60 µg) were resolved on 7.5% or 15% SDS-PAGE and electrophoretically transferred to polyvinylidene difluoride membranes. After blocking, the membranes were incubated overnight at 4 °C with primary antibodies against TNF-α (1:1000; AbD Serotec, Portugal), IL-6 (1:500; AbCam, UK), MMP9 (1:1000; Merck, Alges, Portugal), and phosphorylated p38 (1:1000, Cell Signaling, USA); phosphorylated ERK (1:1000; Cell Signaling, USA); and phosphorylated AKT (1:1000; Cell Signaling, USA), p38 (1:1000; Biolegend, Miraflores, Portugal), ERK (1:5000, Merck, Madrid, Spain), an dAKT (1:1000; Cell Signaling, USA). After incubation, the membranes were washed and incubated for 1 h at room temperature with anti-rabbit (1:5000), anti-mouse (1:10,000), and anti-rat (1:5000) antibodies conjugated with alkaline phosphatase (Santa Cruz Biotechnology, Dallas, TX, USA). Bands were visualized using the enhanced chemifluorescence (ECF) substrate kit (Amersham, UK, GE HealthCare) on the ChemidocTM Touch system (Bio-Rad Laboratories, Hercules, CA, USA) and quantified using Image Lab version 5.2.1 build 11 (Bio-Rad Laboratories). β-actin was used for normalization (1:10,000, Merck, Portugal).

### 4.7. Dihydroethidium Assay

The dihydroethidium assay was performed to detect and measure the production of ROS in skin cryosections as described previously [65]. Briefly, skin cryosections (10 μm thickness) were incubated with 10 μM DHE (Invitrogen, USA) in a humidified dark chamber at 37 °C for 30 min, followed by fixation with 4% PFA in PBS, for 5 min at RT in the dark, and counterstaining with DAPI. The fluorescent images were acquired with the Carl Zeiss LSM 710 confocal microscope with a 200× magnification. The images were acquired using Zen Blue software and analyzed with Fiji software (NIH, USA).

### 4.8. Statistical Analysis

The statistical analysis was performed using GraphPad Prism, version 8 (GraphPad Software, San Diego, CA, USA). Statistical analysis was performed using Student’s *t*-test. A value of *p* < 0.05 was considered statistically significant. The results are presented as mean ± standard deviation (SD). The data on gene expression were normalized to the TATA box binding protein (Tbp) and the protein expression of Western blots was normalized to β-actin and non-phosphorylated proteins.

## 5. Conclusions

DFUs impose a heavy burden on healthcare systems, and therapeutic approaches to prevent their occurrence or to effectively manage them are very limited. Our study revealed the promising effects of BMP7 to enhance diabetic skin wound healing through several mechanisms. BMP7 decreases inflammation via inhibition of the expression of pro-inflammatory cytokines and the number of pro-inflammatory M1 macrophages while inducing M2 macrophage polarization. In addition to the anti-inflammatory effect, BMP7 has an antioxidative effect, decreasing the levels of ROS in diabetic wounds. Together, these effects enhance the skin’s regenerative capacity, demonstrated by the maturation of granulation tissue with the increase in collagen deposition, and the increase in angiogenesis and cell proliferation. These important findings underscore recombinant human BMP7 as a novel candidate for the treatment of skin wounds in diabetes.

## Figures and Tables

**Table 1:** Body weight and blood glucose levels of streptozotocin-induced diabetic mice. Data are presented as mean ± SD.

Mouse Group	Body Weight (g)	Glycaemia (mg/dL)	n
Control	25.7 ± 1.3	445.3 ± 58.1	4
BMP7	25.8 ± 1.6	463.5 ± 55.6	4

**Table 2:** Primer sequences.

Accession No.	Name	Sequence (5′-3′)	Ta (°C)
NM_013684	Tbp	Forward: ACCCTTCACCAATGA CTCCTATG Reverse: TGACTGCAGCAAATCGCTTGG	58
NM_001314054	Il6	Forward: TGGCTAAGGACCAAGACCATCCAA Reverse: AACGCACTAGGTTTGCCGAGTAGA	60
NM_013693	Tnf	Forward: TCCGAATTCAGTGGAGCCTCGAA Reverse: TGCACCTCAGGGAAGAATCTGGAA	60
NM_013599	Mmp9	Forward: CATAGAGGAAGCCCATTACAG Reverse: GATCCACCTTCTGAGACTTCA	58

Legend: Tbp—TATA binding protein; Il6—interleukin-6; Tnf—tumor necrosis factor; Mmp9—matrix metalloproteinase 9; Ta—annealing temperature.

## Data Availability

The original contributions presented in this study are included in the article. Further inquiries can be directed to the corresponding author.
